# Does acupuncture treatment modulate inflammatory cytokines in rodent models of depression? A systematic review and meta-analysis

**DOI:** 10.3389/fnbeh.2024.1329638

**Published:** 2024-01-15

**Authors:** Ziyi Guo, Zhuoyu Ren, Jianping Yao, Yamin Li, Zhiying Che, Zhiyang Yu, Peigang Fang, Xiao Lu, Min Chen

**Affiliations:** ^1^Faculty of Chinese Medicine, Macau University of Science and Technology, Taipa, Macao SAR, China; ^2^State Key Laboratory of Quality Research In Chinese Medicine, Macau University of Science and Technology Taipa, Taipa, Macao SAR, China; ^3^Department of Anesthesiology, Pain and Perioperative Medicine, The First Affiliated Hospital of Zhengzhou University, Zhengzhou, China; ^4^Henan University of Chinese Medicine, Zhengzhou, China; ^5^Zhuhai Campus, Zunyi Medical University, Zhuhai, China

**Keywords:** depression, acupuncture, electroacupuncture, inflammation cytokines, rodent animal model

## Abstract

**Background:**

Despite the increasing global prevalence of depression, existing treatment methods have limitations. Acupuncture has been recognized for its potential to alleviate various diseases by regulating inflammatory cytokines. However, a comprehensive systematic analysis of the effects of acupuncture on depression through inflammatory cytokines is currently lacking. This review aims to evaluate the impact of acupuncture on inflammatory cytokines in animal models of depression.

**Methods:**

A comprehensive search was conducted in PubMed, EMBASE, MEDLINE, and the Research Information Service System to identify studies that met predefined inclusion and exclusion criteria. The quality of each included study was assessed using a 10-item checklist adapted from the Cochrane Collaboration methods and animal data review. Meta-analysis was performed using STATA 17.0 software for literature that met the inclusion criteria.

**Results:**

The meta-analysis included a total of 21 studies involving 376 rodents. The overall quality of the included reports was rated as moderate or higher. The results demonstrated that acupuncture had a significant effect on the reduction of pro-inflammatory cytokines, including: IL-1β [SMD = 3.36, 95% CI (2.73, 4.00), *I^2^* = 73.3%, *p* < 0.05], IL-6 [SMD = 3.05, 95% CI (2.45, 3.64), *I^2^* = 68%, *p* < 0.05], and TNF-α [SMD = 3.30, 95% CI (2.53, 4.06), *I^2^* = 74.5%, *p* < 0.05]. Conversely, acupuncture was associated with an increased expression of anti-inflammatory cytokines, notably: IL-4 [SMD = −1.64, 95% CI (−2.46, −0.82), *I^2^* = 4.1%, *p* = 0.307] and IL-10 [SMD = −1.45, 95% CI (−2.24, −0.66), *I^2^* = 0, *p* = 0.678]. These results suggest that acupuncture modulates cytokine levels in depressed rodents, including reducing the expression of pro-inflammatory cytokines and increasing the expression of anti-inflammatory cytokines, thereby regulating the immune-related antidepressant pathway.

**Conclusion:**

While this study is limited by the number of included studies, the results suggest that acupuncture may be a viable option for the treatment of depression, and this effect is achieved through the regulation of various inflammatory cytokines.

**Systematic review registration:**

This research endeavor was duly registered with PROSPERO (ID: CRD42023420919, https://www.crd.york.ac.uk/PROSPERO/display_record.php?RecordID=420919).

## 1 Introduction

Depression, characterized by diminished interest and a depressed mood, is a prevalent psychiatric disorder often accompanied by physical symptoms, cognitive impairment, and disruptions in volitional activity, eating, and sleeping patterns. According to the World Health Organization, more than 322 million people worldwide are affected by this disorder (GBD 2019 Mental Disorders Collaborators, 2022). Many scholars posit that depression is a complex ailment influenced by multiple factors, including genetic, psychological, and biological components (Liston et al., 2022). Currently, mainstream treatment modalities for depression involve psychotherapies and pharmacotherapies, both of which often entail extended treatment durations and elevated costs. Recent evidence suggests that the efficacy of these therapeutic approaches may have been overestimated (Bertolini et al., 2022; Leichsenring et al., 2022; Dobrek and Głowacka, 2023). In light of these constraints, there is an imperative requirement to ascertain a simple, cost-effective, and efficacious treatment approach for depression.

Acupuncture, originating from China with a rich historical legacy, stands as a therapeutic method offering a myriad of advantages, including simplicity of application, minimal adverse reactions, and cost-effectiveness. Substantiated as a viable treatment for various mental disorders, recent research underscores acupuncture’s significant role in mitigating symptoms of depression (Cutler et al., 2023; Wang et al., 2023; Chen et al., 2023b). Its efficacy is comparable to antidepressant medications, boasting additional merits of economic efficiency, convenience, and reduced adverse reactions (Xu et al., 2023). Peripheral levels of inflammatory markers have been shown to closely correlate with the severity of depression (Suarez et al., 2003; Köhler-Forsberg et al., 2019). Clinical investigations have revealed abnormal levels of inflammatory factors in patients with depression (Ellul et al., 2016). However, multiple meta-analyses consistently demonstrate that acupuncture significantly improves symptoms of various central nervous system disorders, including depression, with proven anti-inflammatory effects (Armour et al., 2019; Xu et al., 2022; Lam Ching et al., 2023). Acupuncture exerts its anti-inflammatory effects through various pathways, including the vagus nerve-adrenal medulla-dopamine pathway, vagus nerve-adrenal axis/spinal sympathetic pathway, vagus nerve-spleen cholinergic pathway, or percutaneous auricular vagus nerve-cholinergic anti-inflammatory pathway. Acupuncture at superficial acupoints induces various somatosensory-autonomic reflex pathways, playing a crucial role in modulating the body’s immune-inflammatory responses (Liu et al., 2021; Yang et al., 2023).

While acupuncture has been substantiated as an effective therapeutic intervention with substantial clinical experience in treating depression, the precise impact of acupuncture on inflammation and immune function remains unresolved. Consequently, there is an urgent need for in-depth preclinical research to investigate whether acupuncture can exert anti-depressant effects through its anti-inflammatory and immune-modulating capabilities. Against this backdrop, a comprehensive statistical evaluation confirming the anti-depressant effects of acupuncture is still lacking.

Answering the aforementioned questions through human clinical studies poses a formidable challenge. However, significant strides have been made in animal research, demonstrating the anti-inflammatory effects of acupuncture and unveiling potential mechanisms for acupuncture in treating depression. To our knowledge, there is currently no literature review documenting the anti-inflammatory mechanisms of acupuncture in the treatment of depression. In this context, we present the first meta-analysis, examining the impact of acupuncture on inflammatory factors in various animal models of depression. We summarize the intervention strategies of acupuncture employed in animal studies and their effects on inflammatory cytokines associated with depression. This study aims to investigate whether acupuncture exerts antidepressant effects by modulating cellular inflammatory factors. Overall, acupuncture may serve as a valuable guide for rehabilitation and offer novel therapeutic strategies to enhance the clinical management of depression.

## 2 Methodology

The meta-analysis was conducted in accordance with the PRISMA guidelines (Moher et al., 2009) and Cochrane Collaboration standards (Cumpston et al., 2019) Furthermore, this research endeavor was duly registered with PROSPERO (ID: CRD42023420919).

### 2.1 Search strategy

The authors rigorously conducted a systematic search across multiple electronic databases, encompassing PubMed, Embase, Google Scholar, Medline, China National Knowledge Infrastructure, Wanfang Database, and Scopus. This exhaustive search encompassed records dating from the inception of these databases up to July 2023. The search strategy involved the use of the following terms: (((depressive [Mesh]) OR (Depressive Disorder [Mesh]) OR (Depressive disorder, Major [Mesh]) OR (Depression [Mesh]) OR (Depressive symptoms) OR (Depressive Symptom)) AND ((Acupuncture [Mesh]) OR (Electroacupuncture [Mesh]) OR (Acupuncture therapy [Mesh]))) AND (((major depression[Title/Abstract]) OR (major depressive disorder[Title/Abstract]) OR (depressive symptom[Title/Abstract]) OR (mood disorder[Title/Abstract]) OR (emotional depression[Title/Abstract])) AND ((auricular acupuncture[Title/Abstract] OR (hand acupuncture[Title/Abstract]) OR (auriculotherapy[Title/Abstract]))). There were no language or publication type restrictions imposed during the search process. Furthermore, a meticulous manual search of the reference lists of the included articles was executed, with the exception of original animal studies, in order to enhance the comprehensiveness of the study’s literature review.

### 2.2 Inclusion/exclusion criteria

Studies were included based on the following criteria: subjects (rodent models of depression), interventions (acupuncture as the primary therapeutic modality, specifically restricted to manual acupuncture and electroacupuncture), and outcomes (with a primary focus on assessing the levels of individual inflammatory cytokines as the principal markers to evaluate the efficacy of acupuncture). Additionally, behavioral data obtained from animal studies were considered secondary outcomes. Studies that did not meet these specific criteria or did not provide access to the full text were excluded. Clinical trials unrelated to the topic of interest were also excluded, and after a thorough examination of the full text, studies that did not align with the established criteria regarding their methodologies and reported results were further excluded from consideration.

### 2.3 Data extraction

Data extraction was carried out independently by two authors, namely GZY and RZY, and encompassed the following variables: publication year, first author’s name, type of rodent depression model, specific disease or condition, sample size, type of acupuncture employed, and the nature of the specimen under investigation. In addition to these parameters, mean values of inflammatory cytokine levels within each group (e.g., disease group, intervention group) were extracted, along with corresponding measures of variability. These data were instrumental in determining effect measures and effect sizes associated with acupuncture’s impact on depression. In instances where data were exclusively presented graphically, concerted efforts were made to procure the necessary measurements. This entailed direct contact with the study authors or the utilization of GetData software to derive the requisite values. Furthermore, secondary outcomes were recorded in the form of behavioral experiment data, encompassing details about the specific type of behavioral test employed and the corresponding results.

### 2.4 Quality assessment

To assess the methodological quality of each included study, a rigorous evaluation was conducted by two authors, namely GZY and RZY. The assessment was based on a 10-item checklist that had been adapted from the Collaborative Approach to Meta-Analysis and Review of Animal Data from Experimental Studies (CAMARADES) checklist (references 14 and 15). The checklist included the following criteria: Publication in a peer-reviewed journal. Statements describing the control of temperature. Random allocation of subjects to treatment or control groups. Blinded construction of the model. Use of animals with hypertension or diabetes, if applicable. Blinded assessment of study outcomes. Utilization of anesthetic without marked intrinsic properties. Sample size calculation. Compliance with animal welfare regulations. Declaration of any potential conflicts of interest.

### 2.5 Statistics

In the context of continuous variables, we computed the standardized mean differences (SMD) along with their corresponding 95% confidence intervals (CI). The assessment of heterogeneity was carried out through the use of the I-square statistic (*I^2^*). In instances where *I^2^* was less than 50%, fixed-effects models were employed; conversely, when *I*^2^ exceeded 50%, random-effects models were utilized. In order to identify potential sources of heterogeneity, we conducted a sensitivity analysis. Subgroup analysis was conducted to investigate the source of heterogeneity and model species, establishment of animal model, the duration of treatment, the timing of treatment, acupuncture method, source of organization. Egger’s test and Begg’s test were used to analyze the publication bias of the selected study. All statistical analyses were performed using STATA13.0 and STAT17.0.

## 3 Results

### 3.1 Research screening

**Figure 1:**
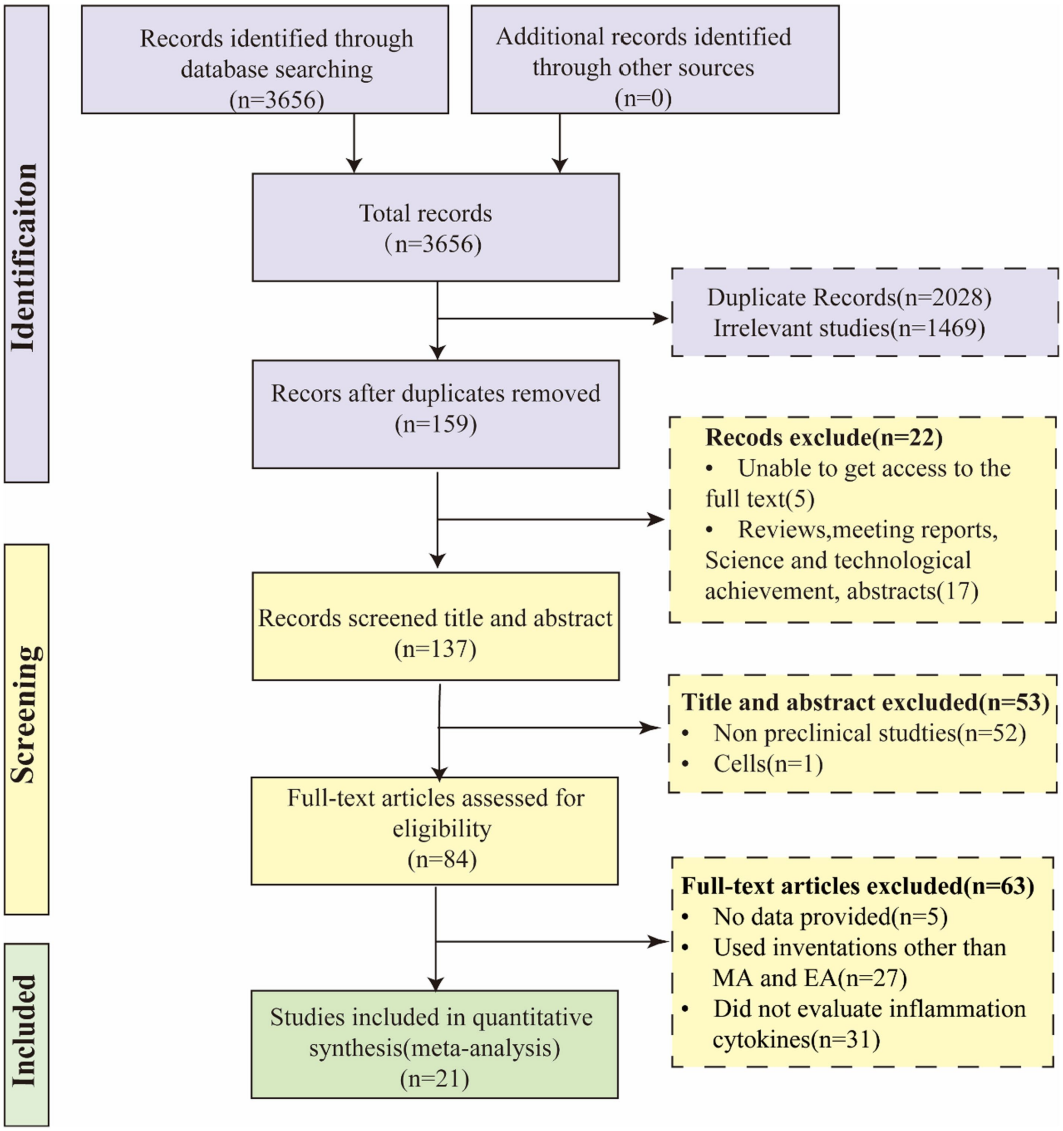
The flowchart of paper inclusion process.

Our research endeavor commenced with the implementation of a retrieval strategy, which yielded a total of 3,656 pieces of data. Following the elimination of duplicates and irrelevant literature, 159 articles remained. Subsequently, we further excluded 17 researches including review articles, meeting reports, scientific and technological achievements, as well as abstracts. In addition, access to full text was unavailable for 5 articles, and these were also excluded, leaving us with 137 studies for continued screening. By evaluating titles and abstracts, we excluded 52 non-preclinical studies and 1 study specifically related to cells. Subsequently, a comprehensive examination of the full text was conducted for 84 articles. Among these, 5 articles met the inclusion criteria but lacked available data, and were thus excluded. Additionally, 27 articles used interventions other than MA and EA and 31 articles did not evaluate inflammation cytokines, leading to their exclusion as well. Ultimately, 21 studies were selected for a comprehensive analysis (Guo et al., 2014, 2020; Jin et al., 2014; Mo et al., 2014; Lu et al., 2016; Kim et al., 2017; Yu et al., 2017; Dong, 2018; Han et al., 2018; Cai et al., 2019; Zhao et al., 2019; Wang et al., 2020, 2022; Zhang et al., 2020; Zhao, 2020; Li X. et al., 2021; Li X. Y. et al., 2021; Chen et al., 2022; Wu et al., 2022; Chen W. et al., 2023; Wei et al., 2023). Figure 1 illustrates the literature screening process.

### 3.2 Research characteristic

**Figure 2:**
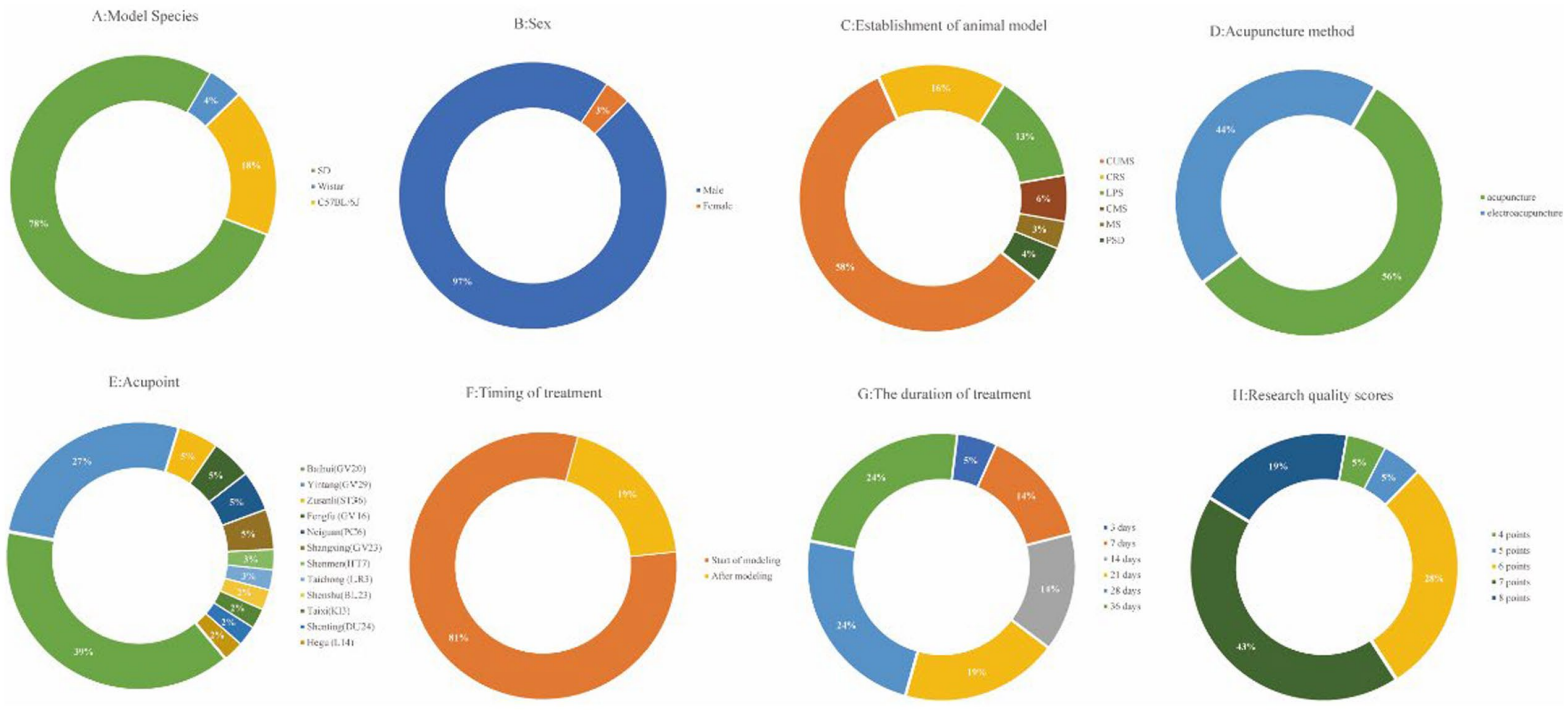
Included study characteristics. **(A)** Model species; **(B)** Sex; **(C)** Establishment of animal model; **(D)** Acupuncture method; **(E)** Acupoint; **(F)**Timing of treatment; **(G)** The duration of treatment; **(H)** Research quality scores.

The basic characteristics of the 21 selected studies are summarized in Table 1. A total of 376 animals were included in this research. The majority of these animals were male Sprague–Dawley rats, accounting for 78% of the sample, followed by C57BL/6 J mouse at 18%, and Wistar rats at 4% (Figure 2A). Notably, 97% of the animals were male, with only 3% being female (Figure 2B). Regarding the depression animal models employed, six distinct models were utilized, with the Chronic Unpredictable Mild Stress (CUMS) model being the most prevalent, featured in 58% of the studies. The Chronic Restraint Stress (CRS) model was employed in 16% of the studies, while the Lipopolysaccharide (LPS) model was found in 13%. Other models included Chronic Mild Stress (CMS) at 6%, Psychosocial Defeat (PSD) at 4%, and Maternal Separation (MS) at 3%. In terms of acupuncture methods, manual needling was the predominant choice in 56% of the studies, while the remaining 44% utilized electroacupuncture (Figure 2C). Furthermore, there was variability in the selection of acupuncture points. Baihui acupuncture point was the most commonly selected (39%), followed by Yintang (27%). Other acupuncture points, such as Zusanli, Fengfu, Neiguan, and Shangxing, were chosen in 5% of the studies. Shenmen and Taichong were the least frequently selected acupuncture points, each appearing in only 2% or 3% of the studies (Figure 2D). In the context of timing and duration of treatment, 81% of the studies implemented acupuncture at the commencement of modeling, while 19% conducted acupuncture treatment after the modeling process (Figure 2E). The duration of acupuncture treatment varied, ranging from 3 to 36 days. Notably, 28-day and 36-day treatment durations were the most common, each representing 24% of the studies. Treatment for 21 days accounted for 19%, while 7-day and 14-day treatments were each employed in 14% of the studies. A 3-day treatment duration was noted in 5% of the studies (Figure 2F).

**Table 1:** Characteristics of included studies.

Studies	Model species (M/E)	Sex	Model	Method	Acupoint	Duration	Timing
Wenjie Chen, 2023	SD (20/20)	Male	CUMS	MA	GV23, GV16	14	Start
Xingying Wu, 2023	C57BL/6 J (16/16)	Male	LPS	EA	L14, LR3	3	After
Qi Wang,2021	C57BL/6 J (20/20)	Male	CMS	EA	GV 20, BL 23, KI3	21	After
Xiaoyan Li,2021	SD (10/10)	Male	CUMS	MA	GV20, GV29	36	Start
Hongmei Wang,2020	SD (16/16)	Male	CUMS	MA	GV20, GV29	36	Start
Xiaoyan Li,2021	SD (16/16)	Male	CUMS	MA	GV20, GV29	36	Start
Kun Zhang,2020	Wistar (16/16)	Male	LPS	EA	GV20, GV29	7	Start
Dongsoo Kime,2017	SD (12/12)	Female	MS	MA	HT 7, BL57	7	Start
Jun Lu,2016	SD (16/16)	Male	CUMS	MA	GV20, PC6	14	Start
Wa Cai,2019	SD (16/16)	Male	PSD	EA	GV20, GV29	21	After
Yaoguo Han,2018	C57BL/6 J (32/32)	Male	LPS	EA	GV20	7	Start
Yiping Chen,2022	SD (18/18)	Male	CUMS	MA	GV23, GV16	14	Start
Tianwei Guo, 2014	SD (20/20)	Male	CUMS	EA	GV20, GV29	21	Start
Xiao Guo, 2020	SD (20/20)	Male	CUMS	EA	ST36	28	After
Gaowen Wei, 2023	SD (16/16)	Male	CUMS	MA	GV20, GV29	36	Start
Shuying Jin, 2014	SD (16/16)	Male	CUMS	MA	GV20, PC6	28	Start
YaZhao, 2021	SD (16/16)	Male	CUMS	MA	GV20, GV29	36	Start
Qiuyun Yu, 2017	SD (40/40)	Male	CRS	MA	GV20, GV29	28	Start
JunZhao, 2019	SD (12/12)	Male	CUMS	EA	GV20, GV29	28	Start
Sha Dong, 2018	SD (16/16)	Male	CRS	MA	GV20, GV29, ST36	28	Start
Xiaofeng Deng, 2014	SD (12/12)	Male	CUMS	EA	GV20, GV29	21	Start

E, experiment; M, model; MA, matual acupuncture; EA, electroacupuncture.

### 3.3 Quality evaluation

**Figure 3:**
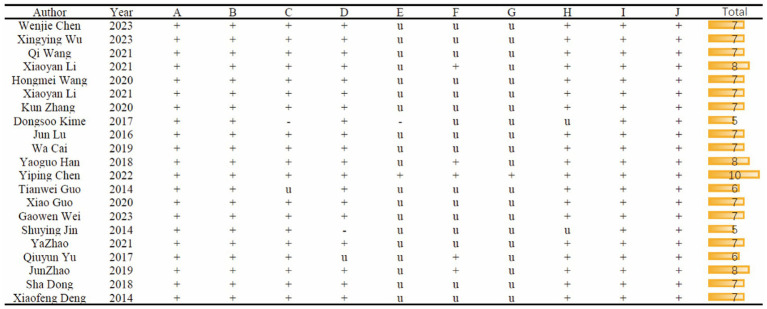
Risk of bias and quality score of included studies. A, Appropriate animal model; B, same size calculation; C, control of temperature; D, random allocation to treatment or model; E, allocation concealment; F, blinded assessment of outcome; G, avoidance of anesthetic with significant intrinsic neuroprotective activity or neurotoxicity; H, compliance with animal welfare regulation; I, statement of potential conflict of interests; J, peer-reviewed published.

Figure 3 provides a detailed overview of the study quality assessment, where the SYRCLE 10-item checklist was employed to evaluate the methodological quality of the 21 included studies. The overall quality of these studies was found to be moderate or higher. However, it is worth noting that one study did not provide a description of experimental temperature control. Additionally, one study did not detail the method of random allocation, and one had an unclear description of the random allocation method. Only one study explicitly mentioned the use of allocation concealment methods. In terms of blinding, five studies reported on its implementation. Furthermore, only one study explicitly stated that no anesthetics with significant intrinsic neuroprotective activity or neurotoxicity were used.

### 3.4 IL-1β

**Figure 4:**
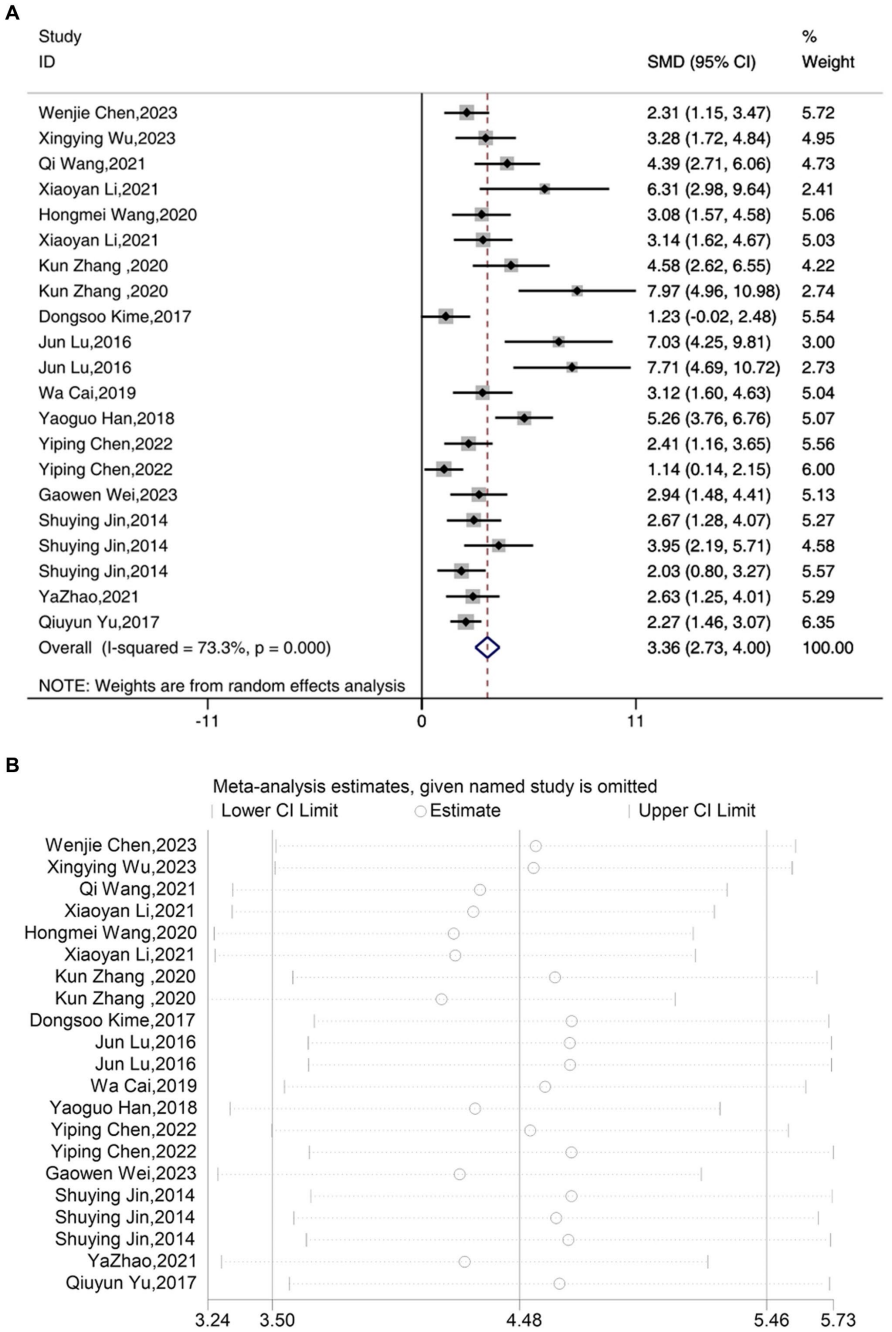
Forest map **(A)** and sensitivity analysis **(B)** of IL-1β.

A total of 17 studies reported the outcome measure IL-1β. The results indicated that the expression levels of IL-1β in the depression model group increased, while after acupuncture treatment, IL-1β levels were significantly lower than those in the depression model group [SMD = 3.36, 95% CI (2.73, 4.00), *I^2^* = 73.3%, *P* < 0.05] (Figure 4A). Sensitivity analysis showed that the combined effect estimates of all studies were within the confidence interval of the overall effect, indicating the reliability of the study results (Figure 4B).

### 3.5 IL-6

**Figure 5:**
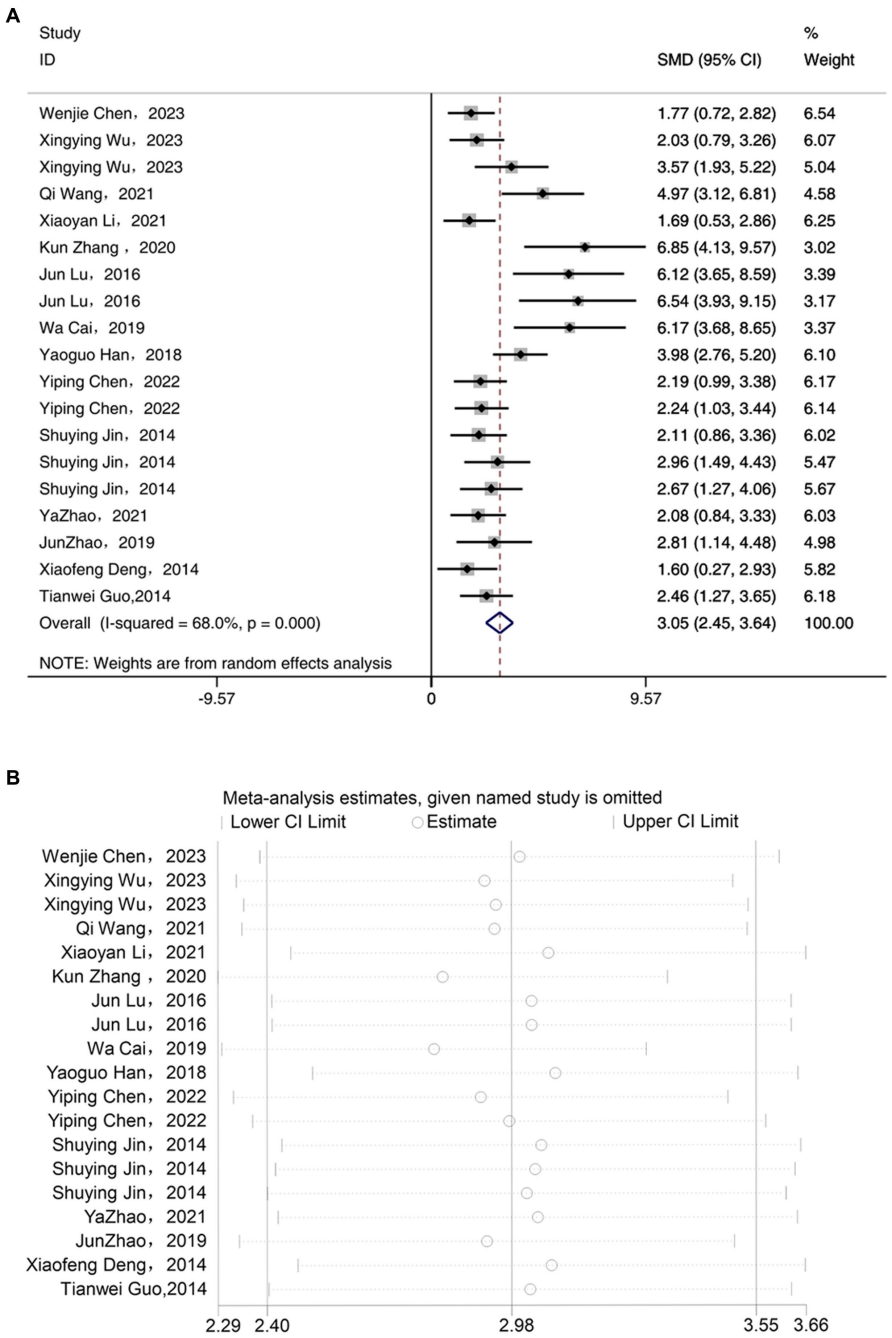
Forest map **(A)** and sensitivity analysis **(B)** of IL-6.

Meta-analysis of 14 studies included demonstrated that acupuncture has an anti-inflammatory effect, significantly reducing the expression levels of IL-6 compared to the depression model group [SMD = 3.05, 95% CI (2.45, 3.64), *I^2^* = 68%, *P* < 0.05] (Figure 5A). Sensitivity analysis showed that the IL-6 results were robust (Figure 5B).

### 3.6 TNF-α

**Figure 6:**
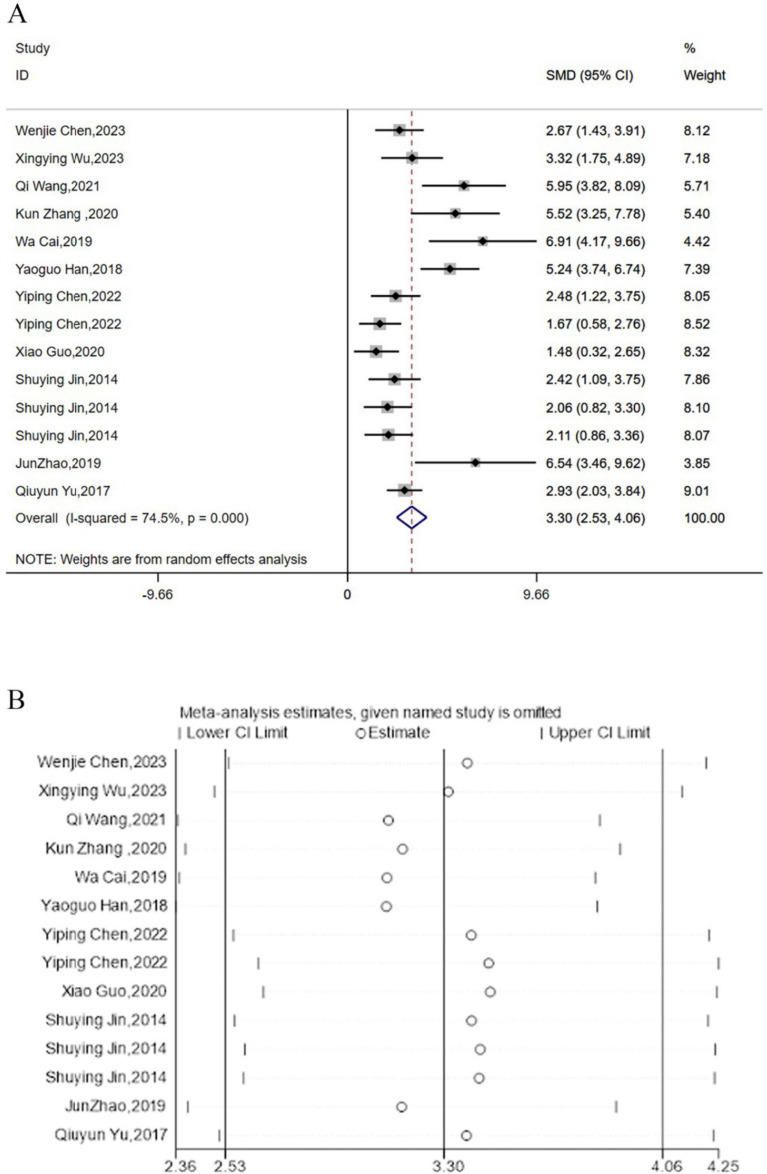
Forest map **(A)** and sensitivity analysis **(B)** of TNF-α.

Twelve studies reported the regulation of TNF-α expression levels by acupuncture. Meta-analysis results indicated that acupuncture could significantly lower the expression levels of TNF-α [SMD = 3.30, 95% CI (2.53, 4.06), *I^2^* = 74.5%, *P* < 0.05] (Figure 6A), and sensitivity analysis confirmed the robustness of the TNF-α conclusion (Figure 6B).

### 3.7 IL-10

**Figure 7:**
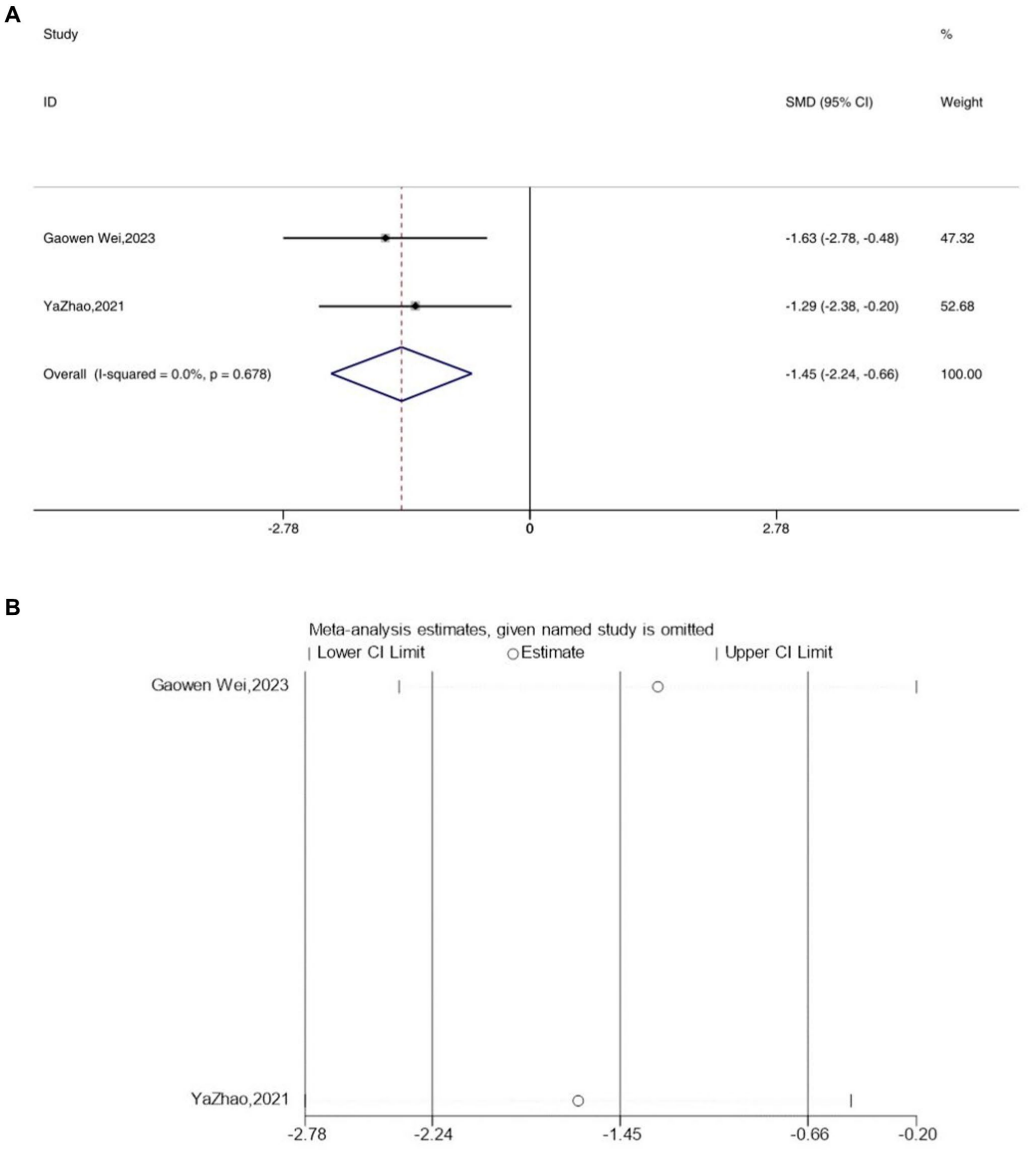
Forest map **(A)** and sensitivity analysis **(B)** of IL-10.

Two studies reported the outcome measure IL-10, and due to an *I*^2^ value of less than 50, a fixed-effects model was applied for the analysis. Interestingly, following acupuncture treatment, a significant reduction in the expression levels of IL-10 was observed in the depressive model mice [SMD = −1.45, 95% CI (−2.24, −0.66), *I^2^* = 0, *p* = 0.678] (Figure 7A). Sensitivity analysis results indicated the reliability of the IL-10 findings (Figure 7B).

### 3.8 IL-4

**Figure 8:**
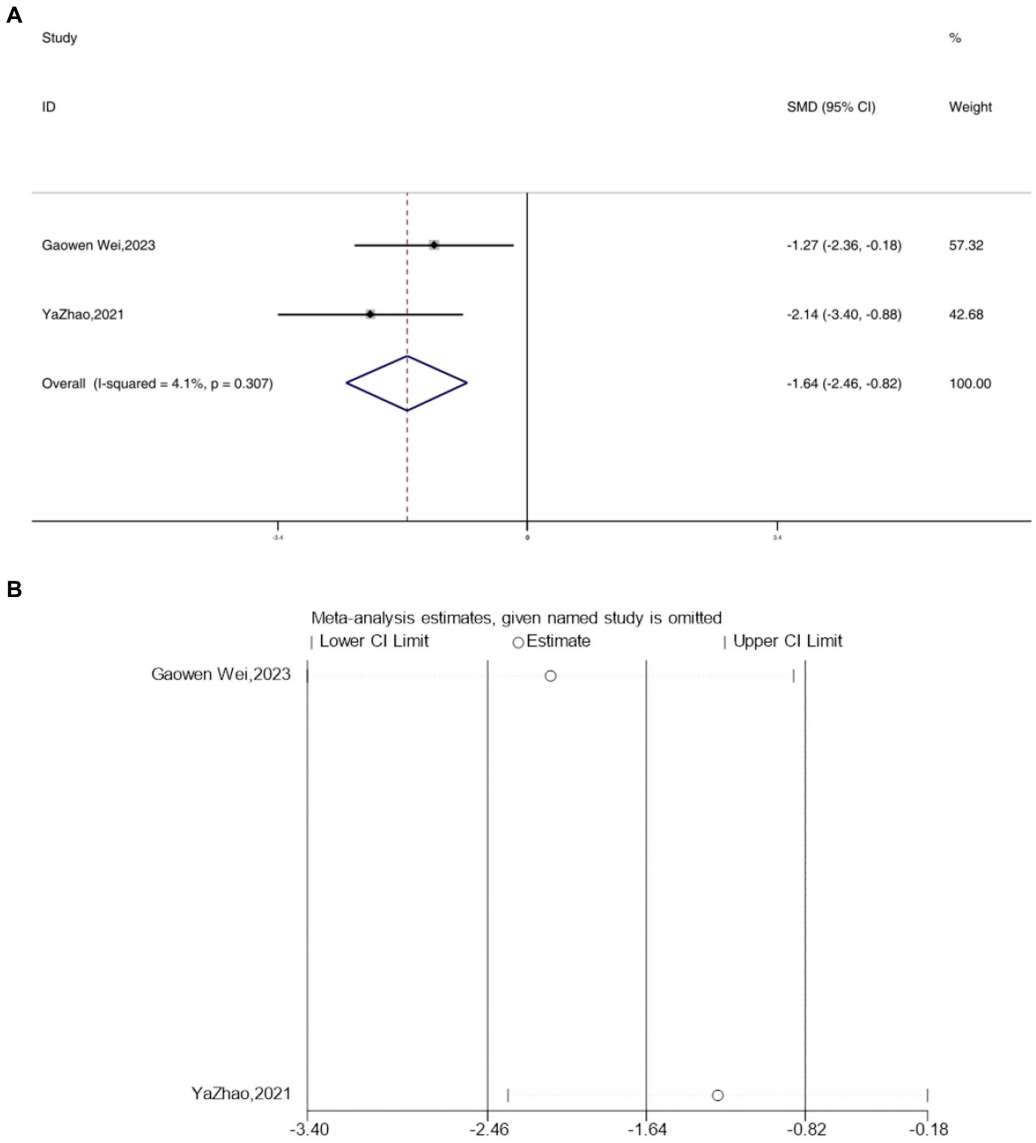
Forest map **(A)** and sensitivity analysis **(B)** of IL-4.

The expression levels of IL-4 were reported in two studies. The analysis using a fixed-effects model indicated a significant decrease in the expression levels of IL-4 in depressive mice IL-4 [SMD = −1.64, 95% CI (−2.46, −0.82), *I^2^* = 4.1%, *p* = 0.307] (Figure 8A). However, following acupuncture treatment, the expression levels of IL-4 increased. Sensitivity analysis confirmed the reliability of the IL-4 results (Figure 8B).

### 3.9 Heterogeneity and results of subgroup analysis

**Figure 9:**
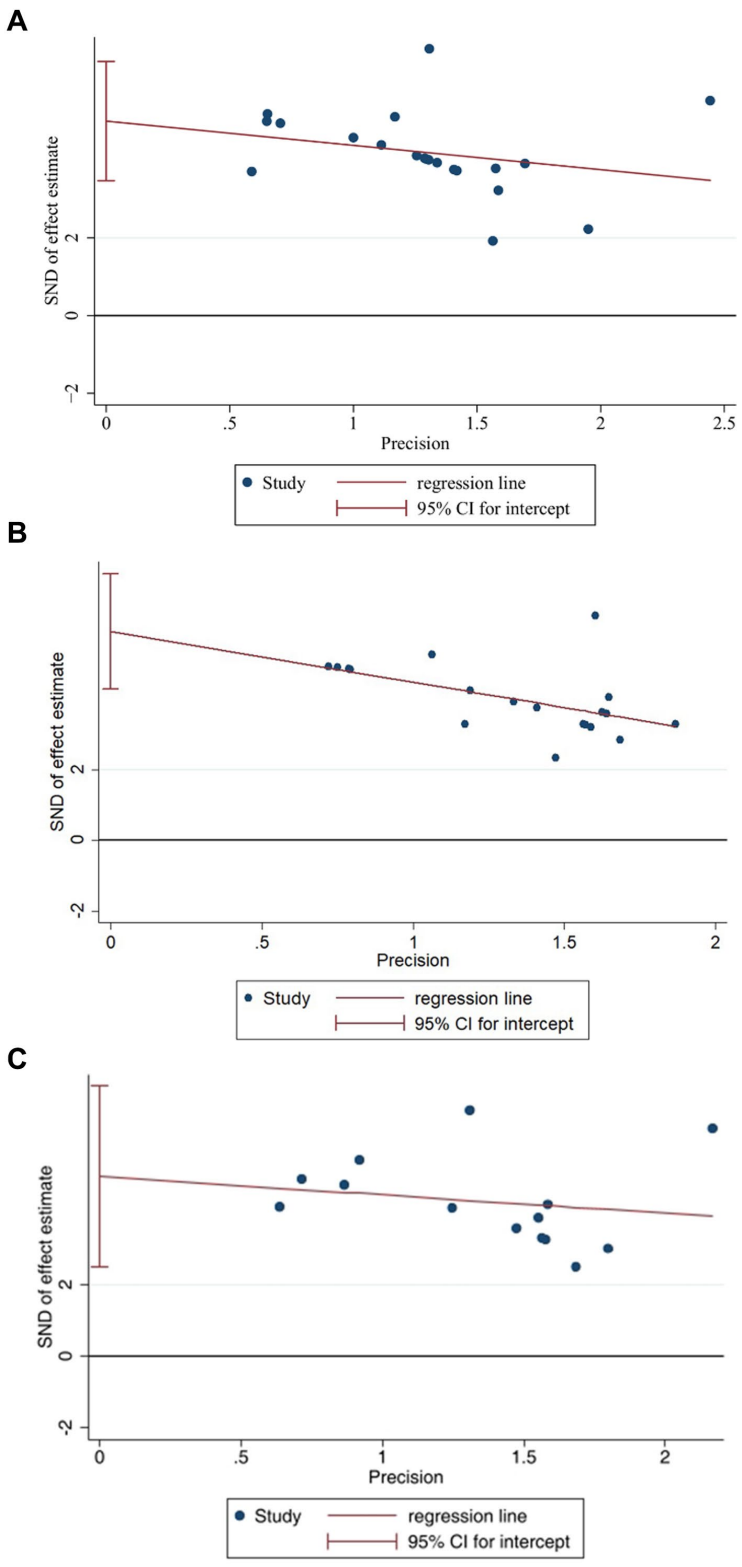
Egger’s test of IL-1β **(A)**, IL-6 **(B)**, TNF-α **(C)**.

We selected the three indicators, IL-1β (Figure 9A), IL-6 (Figure 9B), and TNF-α (Figure 9C), for Egger’s test analysis. The results of Egger’s analysis indicated a certain publication bias within the included studies. Subsequently, we conducted further analysis to explore the potential sources of heterogeneity. Factors such as animal species, acupuncture methods, and timing of acupuncture might be potential contributors to the increased heterogeneity in the results. In this meta-analysis, we employed subgroup analysis to investigate potential evidence for the sources of heterogeneity.

**Figure 10:**
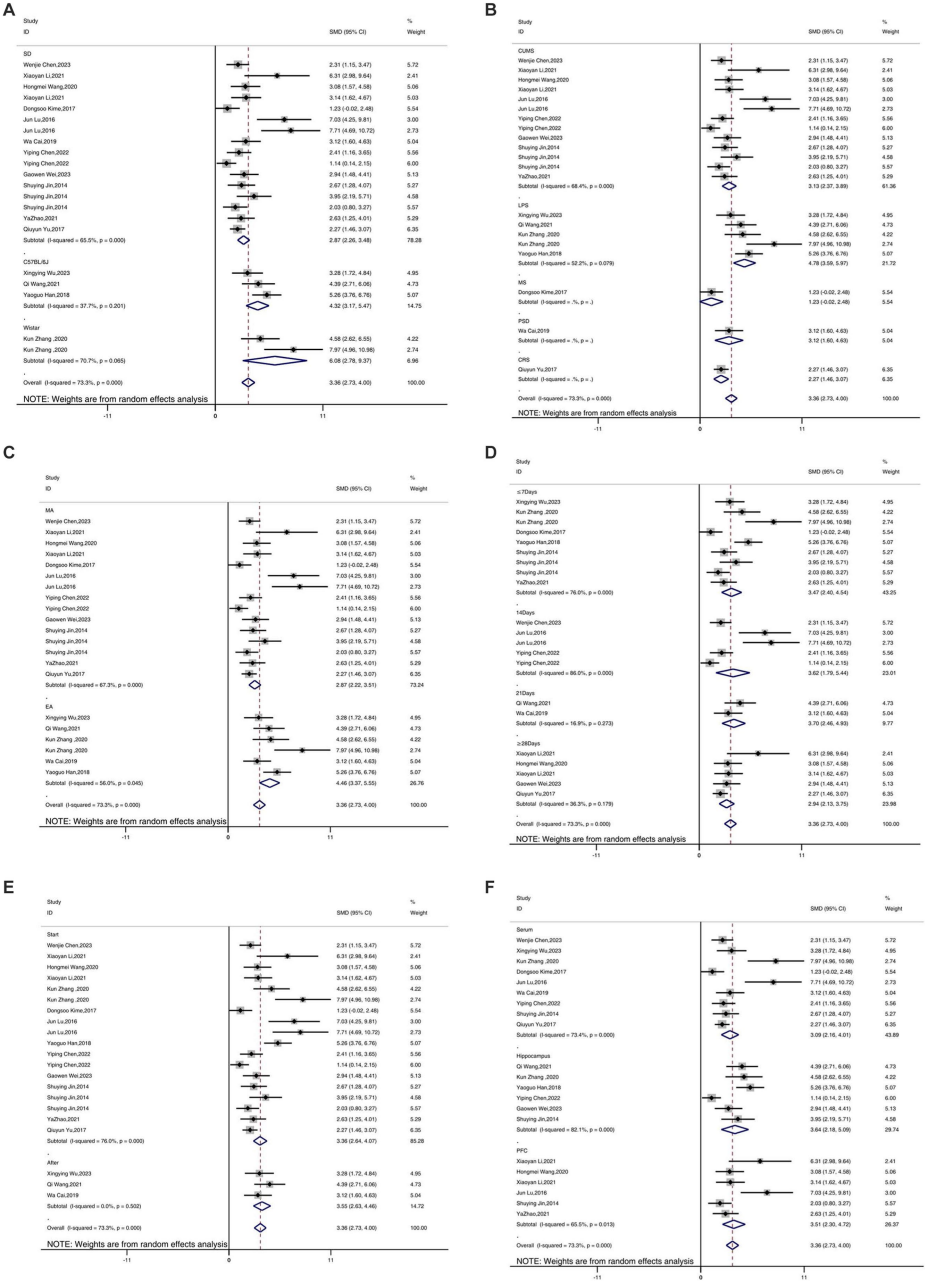
Subgroup analysis of IL-1β. **(A)** Model species; **(B)** Establishment of animal model; **(C)** Acupuncture method; **(D)** The duration of treatment; **(E)** Timing of treatment; **(F)** Resources of organization.

**Figure 11:**
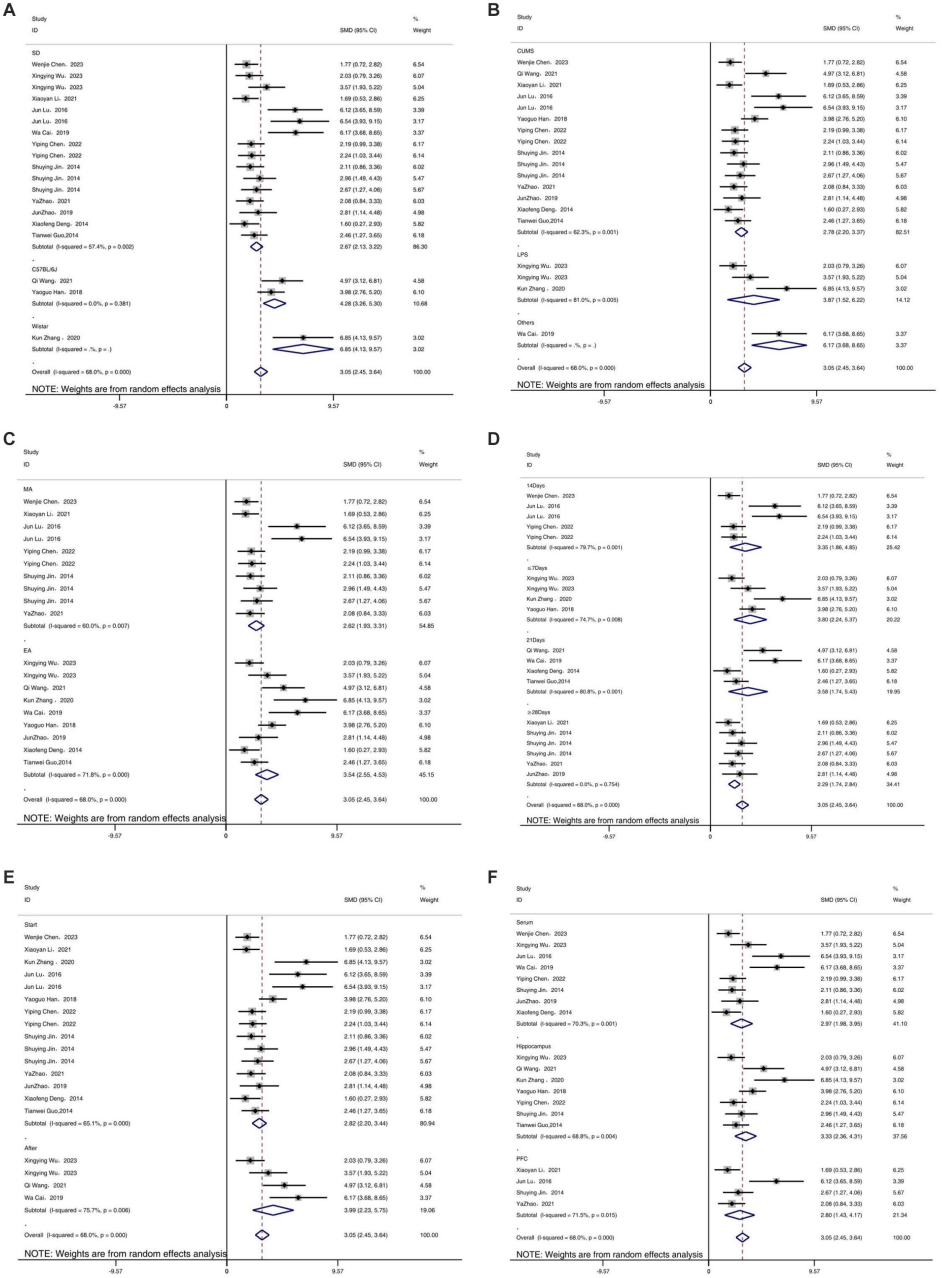
Subgroup analysis of IL-6. **(A)** Model species; **(B)** Establishment of animal model; **(C)** Acupuncture method; **(D)** The duration of treatment; **(E)** Timing of treatment; **(F)** Resources of organization.

**Figure 12:**
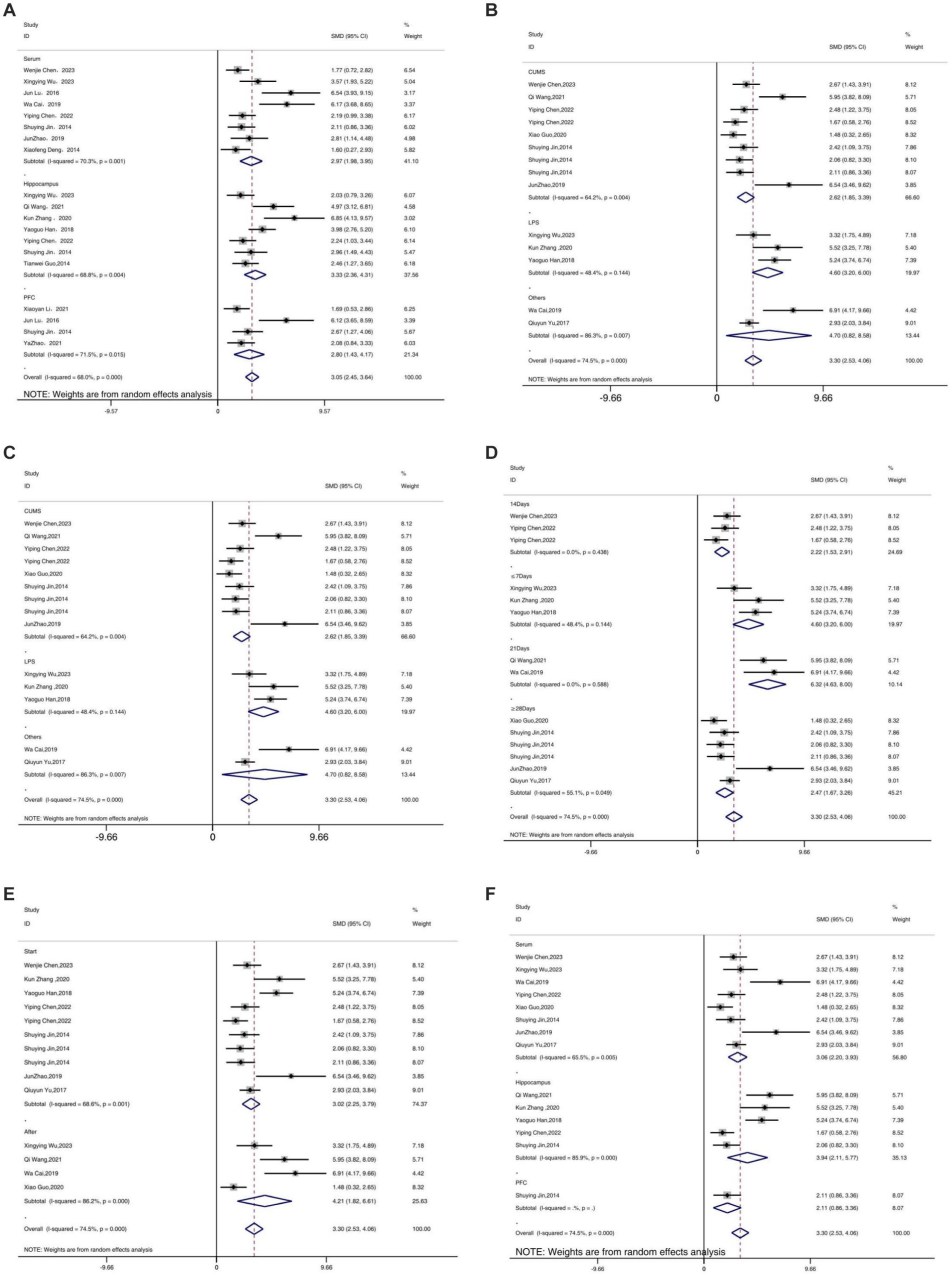
Subgroup analysis of TNF-α. **(A)** Model species; **(B)** Establishment of animal model; **(C)** Acupuncture method; **(D)** The duration of treatment; **(E)** Timing of treatment; **(F)** Resources of organization.

As shown in Figures 10–12, in terms of animal species, only one study was not included in the analysis. Upon analyzing rodents of different species, it was observed that there was considerable heterogeneity. Specifically, for the IL-1β (Figure 10A) and IL-6 indicators (Figure 11A), the heterogeneity significantly decreased in C57BL/6 J mice, showed a slight reduction in SD and Wistar species, but not significantly, suggesting that species may be a source of heterogeneity. The establishment of different animal models did not have a substantial impact on heterogeneity. Additionally, different acupuncture methods were also not found to be a source of heterogeneity. Regarding the timing of acupuncture, the heterogeneity significantly decreased for the IL-1β (Figure 10B) indicator when acupuncture was performed after model establishment, while it increased when performed before model establishment. This suggests that the timing of model establishment may be a potential source of heterogeneity for IL-1β. However, the timing of model establishment did not significantly impact the heterogeneity for IL-6 (Figure 11B) and TNF-α (Figure 12B) indicators. Acupuncture duration also potentially contributed to the observed heterogeneity. For IL-1β (Figure 11D), subgroup analysis showed that when the treatment duration was 21 days or ≥ 28 days, the heterogeneity significantly decreased, but it did not decrease for treatment durations of ≤7 days or 14 days. As for IL-6 (Figure 11D), a treatment duration of ≥28 days resulted in significantly reduced heterogeneity, whereas the other three treatment durations led to an increase in heterogeneity. For TNF-α (Figure 12D), heterogeneity significantly decreased for treatment durations of 14 days and 21 days, while it did not significantly decrease for treatment durations of ≤7 days or ≥ 28 days. This suggests that the acupuncture duration is likely a potential source of heterogeneity. Regrettably, after conducting subgroup analysis, it was found that the different tissue sources had a minimal impact on heterogeneity.

### 3.10 Meta-regression

**Figure 13:**
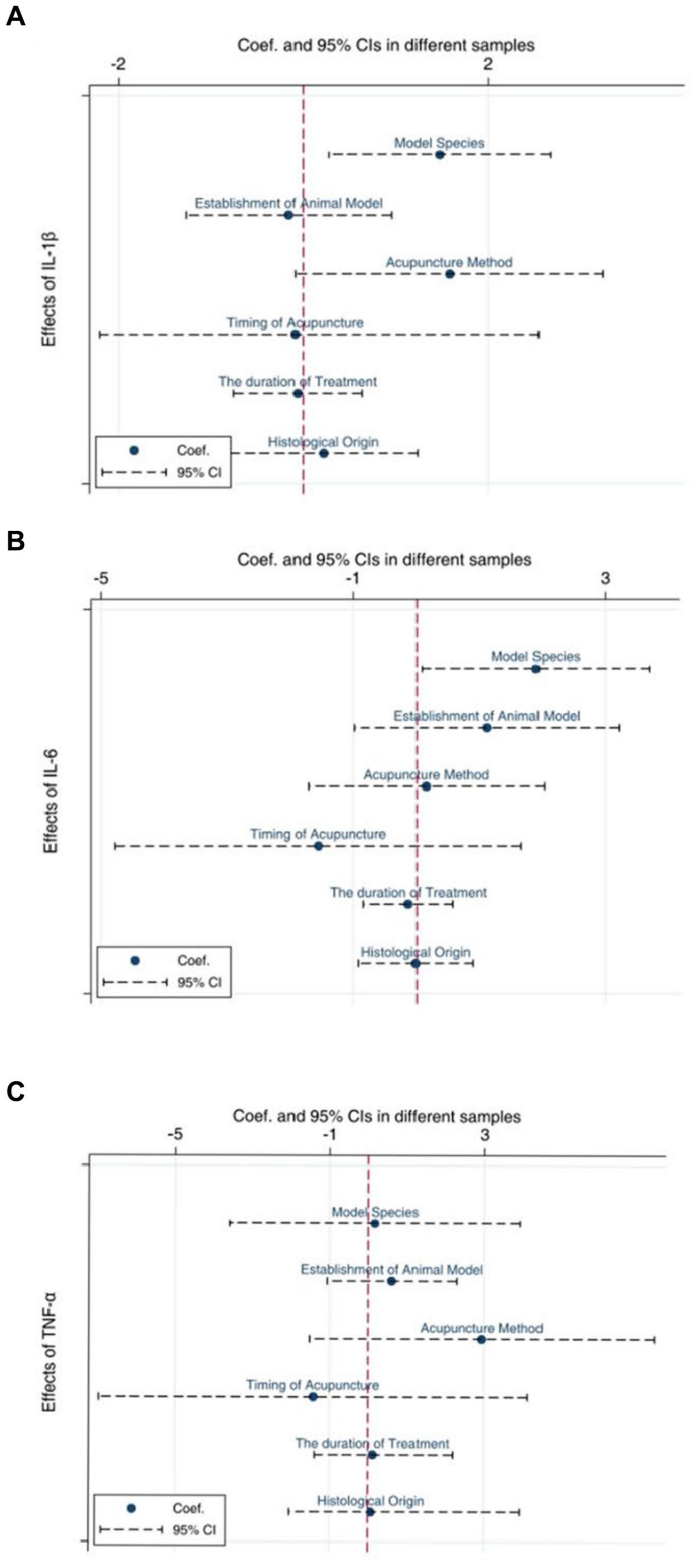
Meta-regression of IL-1β **(A)**, IL-6 **(B)**, TNF-α **(C)**.

In order to explore potential sources of heterogeneity more comprehensively, we conducted the meta-regression analysis. For IL-1 (Figure 13A) and IL-6 (Figure 13B), it appears that species could be a potential source of heterogeneity (*P*<0.05), while other factors did not significantly influence the sources of heterogeneity. However, the regression analysis did not identify any sources of heterogeneity for TNF-α (Figure 13C).

## 4 Discussion

### 4.1 The primary findings

In this review, we systematically gathered information from 21 acupuncture studies utilizing rodent models of depression to determine whether acupuncture can modulate inflammatory cytokines and ameliorate depressive symptoms. Acupuncture or electroacupuncture interventions were applied to rat/mouse models induced by CUMS, LPS, or CRS. Behavioral assessments, including OFT, SPT, FST, TST, EPMT, and NSFT, were employed to evaluate depressive behaviors in the animal models. The results indicate that acupuncture can effectively modulate inflammatory cytokines and improve depressive behaviors in animal models. CUMS is the most commonly used animal model for establishing depression, and acupuncture proves to be an effective intervention for improving depression by modulating inflammatory cytokines. GV20 and GV29 can be considered as fundamental acupoints in animal studies on depression. From the perspective of traditional Chinese medicine, GV20 and GV29 are located on the head and face, belonging to the Governing Vessel meridian. The Governing Vessel is considered the meeting point of all Yang meridians, and acupuncture at these points may regulate the spirit through the Governing Vessel, thereby achieving therapeutic effects for depression. From a modern medical standpoint, stimulating acupuncture points associated with target organs can initiate neuroregulation and exert anti-inflammatory effects by modulating the microenvironment of the acupuncture points. This process may involve the release of neurotransmitters and hormones through the neuroendocrine-immune pathway, ultimately affecting immune cells and regulating the target organs and immune homeostasis of the organism. Different types of acupuncture can modulate various types of cytokines. Moreover, different acupuncture types and point selections demonstrate similar effects in regulating the levels of inflammatory cytokines. The outcomes of meta-analysis reveal a reduction in pro-inflammatory cytokines such as IL-1β, IL-6, and TNF-α following acupuncture. Conversely, anti-inflammatory cytokines like IL-10 exhibit an opposite trend. Therefore, we posit that acupuncture may exert antidepressant effects by regulating anti-inflammatory cytokines and effectively reducing pro-inflammatory cytokines, thereby improving the inflammatory environment.

### 4.2 Implications for future research

Inflammatory cytokines were tested in serum, hippocampus, cortex, and other tissue samples of depression model animals. Inflammatory cytokines can be specifically categorized based on their function: cytokines known to promote inflammation are pro-inflammatory, while those involved in recovery from injury are considered anti-inflammatory cytokines. The results of this meta-analysis show a decrease in pro-inflammatory cytokines such as IL-1β, IL-6, and TNF-α after acupuncture. In contrast, anti-inflammatory cytokines, such as IL-10, exhibited the opposite trend. Thus, we infer that acupuncture can improve the inflammatory environment by inducing anti-inflammatory cytokines and effectively reducing pro-inflammatory cytokines, depending on their mechanisms of depression.

After reviewing animal studies on the mechanism of acupuncture’s antidepressant effects, we have identified several limitations in the current experimental designs. Firstly, the focus of mechanistic research primarily revolves around the brain, with relatively fewer investigations into upstream pathways, especially the central integration mechanisms. Secondly, a significant portion of existing research connects acupuncture’s antidepressant effects with anti-inflammatory responses, while studies exploring the fundamental relationship between acupuncture and immune suppression remain scarce, leaving the precise mechanisms underlying acupuncture’s immune-modulating effects unclear. Thirdly, clinical depression is typically treated after the onset of symptoms. Animal studies suggest that acupuncture can alleviate depression both before and after its onset, but it remains uncertain whether the involved signal transduction mechanisms are similar or distinct. Presently, this field of research lacks comprehensive reports and is fragmented, making it difficult to systematically elucidate the mechanisms involved. Fourthly, the complexity of human depression presents challenges in fully replicating it in clinical practice, given its susceptibility to multiple influencing factors. Consequently, improving animal models and conducting clinical research is necessary to explore acupuncture’s efficacy in depression treatment. Undeniably, our research also has certain limitations. There is heterogeneity in the design of animal research and clinical trials. Subgroup analyses can be performed based on factors such as animal species, modeling methods, acupuncture techniques, the number of acupuncture sessions, the timing of initiation, and tissue sources to investigate potential sources of heterogeneity. However, even after a systematic examination of these factors, the same sources of heterogeneity cannot be definitively identified. Some shortcomings in animal experimental studies have been identified. Firstly, rigor and scientific robustness in study design, execution, analysis, and reporting are crucial for animal research. Nevertheless, the studies included in our analysis suffer from validity issues, particularly in the implementation of acupuncture blinding methods, which could affect the assessment of acupuncture effects. Secondly, existing animal models cannot fully replicate all pathological features of human depression, and differences between various animal models may contribute to heterogeneity. Moreover, considering that women have a higher prevalence of depression compared to men, the gender of animals in experiments should be systematically considered, but few experiments address this aspect. Thirdly, the individualization of traditional Chinese medicine treatments presents challenges for standardizing acupuncture points and electroacupuncture frequencies for different patients, complicating scientific research in acupuncture. Therefore, it is recommended that future research standardizes the procedures of animal experiments, takes into account risk factors such as gender, and explores the effectiveness of different acupoints and parameters in treating depression.

### 4.3 Strengths and limitations of the research

Our study, to the best of our knowledge, represents the first comprehensive meta-analysis discussing acupuncture’s modulation of inflammatory cytokines in rodent models of depression. Our research provides scientific evidence for the current understanding of acupuncture’s regulatory effects on inflammatory cytokines in depression. Firstly, it offers insights into the selection of key acupoints, animal models, and treatment durations, serving as a reference for experimental design in preclinical animal studies. Secondly, acupuncture demonstrates its antidepressant effects by modulating inflammatory cytokines through various inflammatory signaling pathways. Thirdly, this paper provides an initial synthesis of acupuncture’s modulation of inflammatory cytokines in depression.

However, the study has certain limitations. Firstly, cytokines were not classified in a disease-specific manner, as the pathogenesis of depression remains unclear, and the relationship between inflammatory cytokines and depression is yet to be conclusively established. Secondly, the quantity of studies included may not facilitate a comprehensive systematic evaluation. We excluded studies published in languages other than English or Chinese, potentially limiting a thorough understanding of acupuncture’s role in the treatment of depression. While our study has its scope limitations, the analysis of inflammatory cytokines allows for the first assessment of acupuncture’s efficacy in treating depression, which holds meaningful implications.

## 5 Conclusion

In summary, this review has demonstrated that acupuncture treatment may potentially ameliorate depressive behavior in individuals with depression by regulating inflammatory cytokines. The results indicate that acupuncture can improve depressive-like behaviors by modulating both pro-inflammatory and anti-inflammatory cytokines. We anticipate further research on the therapeutic effects of acupuncture and hope that this review serves as a valuable reference in this field of study.

## Data availability statement

The raw data supporting the conclusions of this article will be made available by the authors, without undue reservation.

## Author contributions

ZG: Conceptualization, Data curation, Investigation, Software, Writing – original draft, Writing – review & editing. ZR: Conceptualization, Data curation, Investigation, Software, Writing – review & editing. JY: Funding acquisition, Supervision, Writing – review & editing. YL: Data curation, Writing – review & editing. ZC: Funding acquisition, Writing – review & editing. ZY: Data curation, Writing – review & editing. PF: Data curation, Writing – review & editing. XL: Resources, Writing – review & editing. MC: Writing – review & editing.
